# Therapeutic Effects of Human Umbilical Cord-Derived Mesenchymal Stem Cells in Acute Lung Injury Mice

**DOI:** 10.1038/srep39889

**Published:** 2017-01-04

**Authors:** Hua Zhu, Yi Xiong, Yunqiu Xia, Rong Zhang, Daiyin Tian, Ting Wang, Jihong Dai, Lijia Wang, Hongbing Yao, Hong Jiang, Ke Yang, Enmei Liu, Yujun Shi, Zhou Fu, Li Gao, Lin Zou

**Affiliations:** 1 Pediatrics Research Institute, Ministry of Education Key Laboratory of Child Development and Disorders, Children’s Hospital of Chongqing Medical University, Chongqing 400014, China; 2 Department of Pediatrics, First Affiliated Hospital of China Medical University, Shenyang 110001, China; 3 Department of Respiratory Medicine, Children’s Hospital of Chongqing Medical University, Chongqing 400014, China; 4 Department of Otorhinolaryngology, Children’s Hospital of Chongqing Medical University, Chongqing 400014, China; 5 Chongqing Engineering Research Center of Stem Cell Therapy, Chongqing 400014, China; 6 Laboratoryof Pathology, West China Hospital, Sichuan University, Chengdu 610041, China; 7 Center for Clinical Molecular Medicine, Children’s Hospital of Chongqing Medical University, Chongqing 400014, China

## Abstract

The incidence and mortality of acute lung injury (ALI)/acute respiratory distress syndrome (ARDS) are still very high, but stem cells show some promise for its treatment. Here we found that intratracheal administration of human umbilical cord-mesenchymal stem cells (UC-MSCs) significantly improved survival and attenuated the lung inflammation in lipopolysaccharide (LPS)-induced ALI mice. We also used the proteins-chip and bioinformatics to analyze interactions between UC-MSCs treatment and immune-response alternations of ALI mice. Then we demonstrated that UC-MSCs could inhibit the inflammatory response of mouse macrophage in ALI mice, as well as enhance its IL-10 expression. We provide data to support the concept that the therapeutic capacity of UC-MSCs for ALI was primarily through paracrine secretion, particularly of prostaglandin-E2 (PGE2). Furthermore, we showed that UC-MSCs might secrete a panel of factors including GM-CSF, IL-6 and IL-13 to ameliorate ALI. Our study suggested that UC-MSCs could protect LPS-induced ALI model by immune regulation and paracrine factors, indicating that UC-MSCs should be a promising strategy for ALI/ARDS.

Acute lung injury (ALI), as the early and basic pathophysiologic change in acute respiratory distress syndrome (ARDS), is a challenging disease in clinical critical care medicine^1^. ALI/ARDS is a form of severe respiratory disorders which are most commonly caused by sepsis, pneumonia, trauma and aspiration^2^. Although many ALI/ARDS treatments have been investigated, the mortality is between 36–44% with little improvement^3^. In the United States, it is estimated that there are 190,600 cases and 74,000 deaths annually from ALI that results in a high cost of health-care^4^. However, the current therapeutic strategies for ALI are mainly focused on supportive treatment, and a novel effective therapy is required.

Stem cells, especially mesenchymal stem cells (MSCs), have demonstrated therapeutic potential in lung diseases^5^. MSCs are collected from bone marrow (BM), umbilical cord (UC), adipose tissue, amniotic fluid, skeletal muscle, synovial, gingiva and other tissues^6^. The bone marrow-derived mesenchymal stem cells (BM-MSCs) have presented ability in resolving lipopolysaccharide (LPS)-induced ALI, hyperoxia, pneumonia and systemic sepsis in animal models^7^. However, the clinical application of BM-MSCs is limited due to invasive sample collection, reduced cell number, proliferation, and differentiation capacity in aging donors^8^. Human umbilical cord-derived mesenchymal stem cells (UC-MSCs) are considered a better choice of MSCs for clinical application because of their easy collection, high cell vitality^9^, low immunogenicity^10^, and high paracrine potential for accelerating injury tissue repair processes^11^. UC-MSCs have potential efficacy in the prevention or treatment of lung injury^12^,^13^. However, the therapeutic effects and potential molecular mechanism of UC-MSCs in an ALI model remain unclear. In this study, we found therapeutic effects of UC-MSCs in classical LPS-induced ALI mice by secreting factors.

## Methods

### Animal

All the protocols were carried out in accordance with the approved guidelines. Eight- to ten-week-old BALB/c and C57BL/6 female mice were obtained from the Experimental Animal Center of Chongqing Medical University. All the experiments were approved by the Ethics Committee of Chongqing Medical University.

### Harvesting and concentrating of UC-MSCs conditional medium

When the cell count of UC-MSCs in T25 culture flasks reached 0.5 × 10^6^, the cells were washed 3 times with PBS and re-cultured with 5 ml new serum-free medium for 24 h. Subsequent serum-free medium was used as UC-MSCs conditional medium. For *in vivo* experiments, 2 ml of this original medium was concentrated to an 80 μl volume using a 3000 Da centrifugal filter (Millipore, Massachusetts, USA) by a relative slower centrifugal speed 4500 g for 35–40 minutes, which also could avoid an excessive loss of the small molecule PGE2 in UC-MSCs concentrated medium (Suppl. Fig. 4g).

### Treatment of LPS-induced ALI mice with UC-MSCs or UC-MSCs conditional medium

After 1 hour of LPS exposure, BALB/c or C57/BL6 mice were intravenously administered UC-MSCs (0.5 × 10^6^), or concentrated conditional medium (80 μl, harvested from 0.5 × 10^6^ UC-MSCs), or PBS (80 μl) respectively. Mice were then monitored for post-exposure LPS for 120 h.

### Protein-array of bronchoalveolar lavage fluid (BALF)

Protein-array analysis of BALF was performed using the Raybiotech^TM^ 308 biotin label-based mouse antibody array (Raybiotech, Georgia, USA) according to the manufacturer’s protocol, as the previous report^14^. The expression levels for each protein were normalized to the mean intensity of positive control in all samples and were further analyzed using a fold change ≥2.0 (t-test, two-tailed, *p* < 0.05). Differentially expressed proteins were determined by Hierarchical Clustering (Gene Cluster 3.0, Tokyo, Japan) and gene-ontology (GO) classifications analysis using the DAVID online database.

### Celecoxib inhibited PGE2 synthesis in UC-MSCs

To identify the role of US-MSCs paracrine factor PGE2, we used celecoxib (Pfizer, New York, USA) to block the PGE2 synthesis in cells as previously described^15^. US-MSCs were cultured with different concentrations of celecoxib or control (0.1% DMSO) in 10% FBS DMEM/F12 for 48 h. The cells were then washed with PBS three times and were harvested as celecoxib-pretreated UC-MSCs (UC-MSCs^−PGE2^), in which PGE2 synthesis was inhibited. Alternatively, the UC-MSCs^−PGE2^ were re-cultured with new serum-free medium for 24 h, and the subsequent serum-free medium was used as the PGE2-reduced UC-MSCs conditional medium (CM^−PGE2^). ELISA test was used to analyze the efficacy of celecoxib to inhibit the PGE2 paracrine of UC-MSCs, and MTT assay was used to investigate the effect of celecoxib on the growth of UC-MSCs.

### The investigations of paracrine activities of UC-MSCs

0.5 × 10^6^ UC-MSCs were cultured in 2 ml ALI mice bronchoalveolar lavage fluid (BALF), added with 2 ml DMEM/F12 for 24 h, and cytokines/chemokines secreted by UC-MSCs in culture supernatant were tested by Multiplex-ELISA arrays (Millipore, Massachusetts, USA) according to the manufacturers’ instructions^16^.

### Data and statistical analysis

The values are expressed as the mean ± SE. Multiple comparisons of parametric data were performed using one-way ANOVA at specific time-points (72 h) and two-way ANOVA at different time-points (0 h, 24 h, 72 h, 120 h), followed by Bonferroni multiple test between groups. The survival curves were compared with a log-rank test (Graphpad Prism5.0, California, USA). p < 0.05 was considered statistically significant.

(The other detailed Supplementary Information for Methods is presented online).

## Results

### UC-MSCs improve survival and attenuate lung inflammation in LPS-induced ALI mice

**Figure 1:**
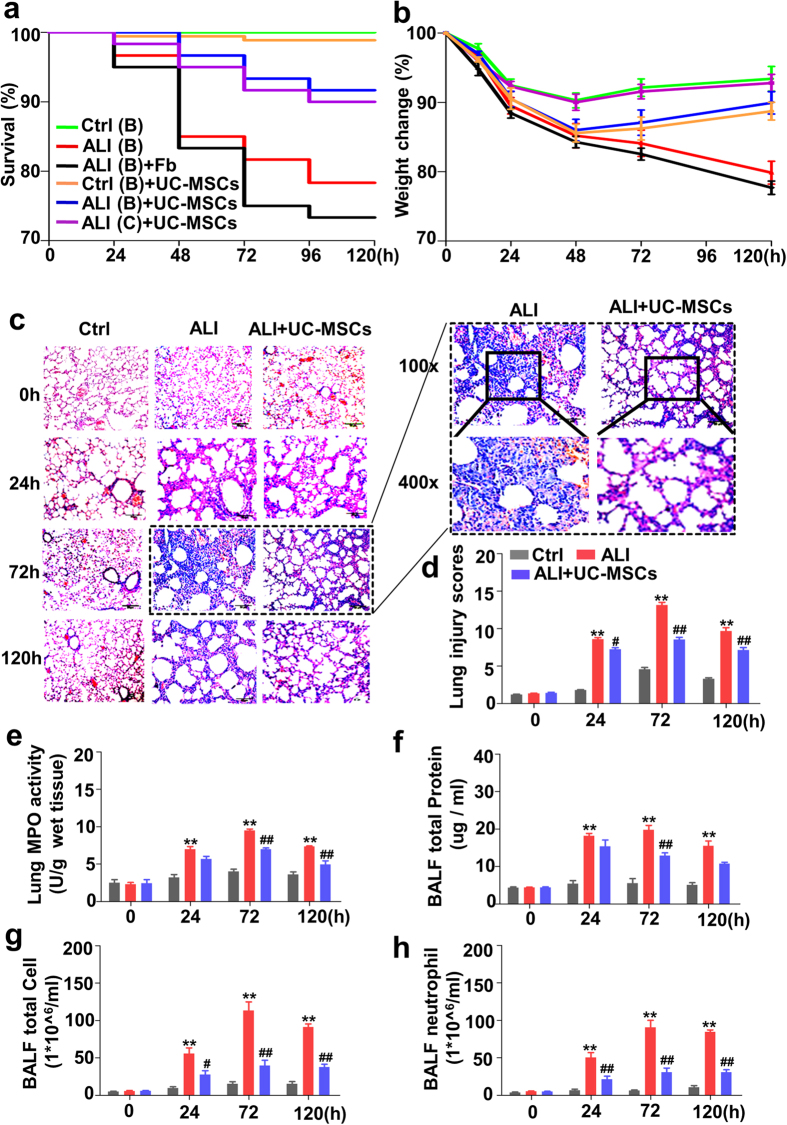
Human umbilical cord-derived mesenchymal stem cells (UC-MSCs) alleviate lipopolysaccharide (LPS)-induced acute lung injury (ALI) mice by intratracheal delivery. Kaplan-Meier survival curves (**a**) and body weight alterations (**b**) of LPS-induced ALI mice after intratracheal delivery of 0.5 × 10^6^ UC-MSCs or fibroblasts (Fb) for 120 h. Green line, BALB/C mice treated with PBS (Ctrl); Red line, BALB/C mice induced by LPS; Black line, LPS-induced BALB/C mice treated with fibroblasts; Orange line, BALB/C mice treated with UC-MSCs; Blue line, LPS-induced BALB/C mice treated with UC-MSCs; Purple line, LPS-induced C57B/L mice treated with UC-MSCs. **(c)** Hematoxylin-eosin (HE) staining of lung sections of BALB/C mice with intratracheal LPS administration or with intratracheal LPS and UC-MSCs at different post-injection times. The lungs from LPS-induced mice treated with UC-MSCs exhibited decreased intra-alveolar edema. Magnifications ×100 or ×400. In (**a**) to (**c**), n = 24 for each group. (**d**) Lung injury scores of LPS-induced ALI mice with or without UC-MSCs treatment. The detection of myeloperoxidase (MPO) activity (**e**), total protein concentration (**f**), total cell count (**g**) and neutrophil count (**h**) in bronchoalveolar lavage fluid (BALF) in LPS-induced ALI mice with UC-MSCs treatment. In (**d**) to (**h**), data are the mean ± SEM, n = 8–10 at each time point. *p < 0.05 or **p < 0.01 *vs.* control mice (Ctrl); ^#^*p* < 0.05 or ^##^*p* < 0.01 *vs.* LPS-induced ALI mice (ALI).

We successfully isolated UC-MSCs from human Wharton’s Jelly (Suppl. Fig. 1) and constructed a stable ALI animal model by intratracheal injection of 5 mg/kg LPS (Suppl. Fig. 2). The ALI mice were then transtracheally injected with UC-MSCs (0.5 × 10^6^) after being administered LPS for 1 h (Suppl. Fig. 2a). The survival and weight improvement of LPS-induced ALI mice treated with UC-MSCs, both in BALB/c and C57/BL6 mice (Fig. 1a,b), indicated the significant therapeutic effects of UC-MSCs in ALI. Then we used BALB/c mice in the following experiments. The pathological damage of lung tissue from LPS-induced ALI mice treated with UC-MSCs was significantly decreased (Fig. 1c,d), especially 72 h post-treatment. The lung injury was evidently declined, meanwhile the myeloperoxidase (MPO) activity, total protein concentration, total cell and neutrophil counts in bronchoalveolar lavage fluid (BALF) were significantly reduced, confirming that UC-MSCs attenuate the lung inflammation of LPS-induced ALI mice (Fig. 1e–h).

### UC-MSCs regulate the lung immune response of LPS-induced ALI mice

**Figure 2:**
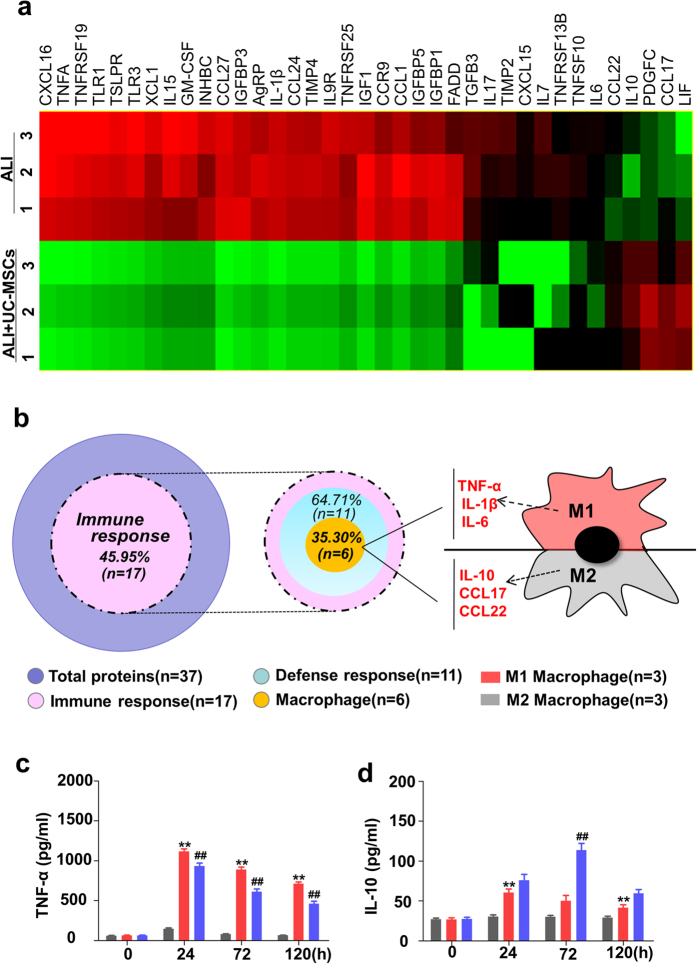
UC-MSCs regulate the protein expression profiles of BALF in LPS-induced ALI mice. (**a**) The heat map of the altered protein files over twofold (*p* < 0.05) in 308 mouse proteins from BALF in LPS-induced ALI mice treated with UC-MSCs for 72 h by cluster analysis (n = 3 each group). Only 32 proteins were significantly decreased and 5 related proteins were significantly increased. The increased proteins are shown in progressively brighter shades of red, and decreased proteins are shown in progressively darker shades of green. (**b**) The analysis of gene-ontology (GO) classifications on 37 differentially expressed proteins. It showed that 17 proteins (45.95%) had a correlation with immune response, and among these 17 proteins, 11 proteins (64.71%) were related to defense response, particularly 6 proteins (35.30%) including TNF-a, IL-1β, IL-6, IL-10, CCL17 and CCL22, were associated with the immune subtypes of macrophages. M1 macrophage, classically activated macrophage. M2 macrophage, alternatively activated macrophage. In (**c)** to (**d**), the concentration of TNF-α and IL-10 in BALF, detected by ELISA from LPS-induced ALI mice treated with UC-MSCs. In (**b**) to (**d**), values are the means ± SE. n = 8 for each group. *p < 0.05 or **p < 0.01 *vs.* control mice (Ctrl); ^#^*p* < 0.05 or ^##^*p* < 0.01 *vs.* LPS-induced ALI mice (ALI).

To understand the molecules involved in US-MSCs treatment for LPS-induced ALI mice, we used a 308 protein array to analyze in the BALF (Suppl. Table 1). Only 32 proteins were significantly decreased, while 5 proteins were significantly increased in the LPS-induced ALI mice treated with UC-MSCs for 72 h (Fig. 2a). The gene-ontology (GO) classifications of these 37 proteins (Suppl. Table 2) showed that 17 proteins (45.95%) had a correlation with the host immune response. Among these 17 proteins, eleven (64.71%) were related to defense response and particularly 6 (35.30%) were associated with the immune subtypes of macrophages (Suppl. Fig. 3a), indicating that UC-MSCs regulate the lung immune response of LPS-induced ALI mice (Fig. 2b).

We also found that the markers of classically activated macrophages (M1, CM), TNF-α, IL-1β and IL-6 were significantly down-regulated by UC-MSCs treatment^17^. More interestingly, in 5 up-regulated proteins, 3 proteins including IL-10, CCL17 and CCL22 were reported to the markers of alternatively activated macrophages (M2, AM)^18^, especially IL-10 had been proved to be a potent anti-inflammatory cytokine to protect lung injury^19^. The results of the q-PCR (Suppl. Fig. 3b) and ELISA assays (Fig. 2c,d) were consistent with those of the protein microarray, suggesting that UC-MSCs treatment would polarize the lung macrophages into M2 macrophages in ALI mice.

### UC-MSCs modulate polarization of mouse macrophages *in vivo* and *in vitro*

**Figure 3:**
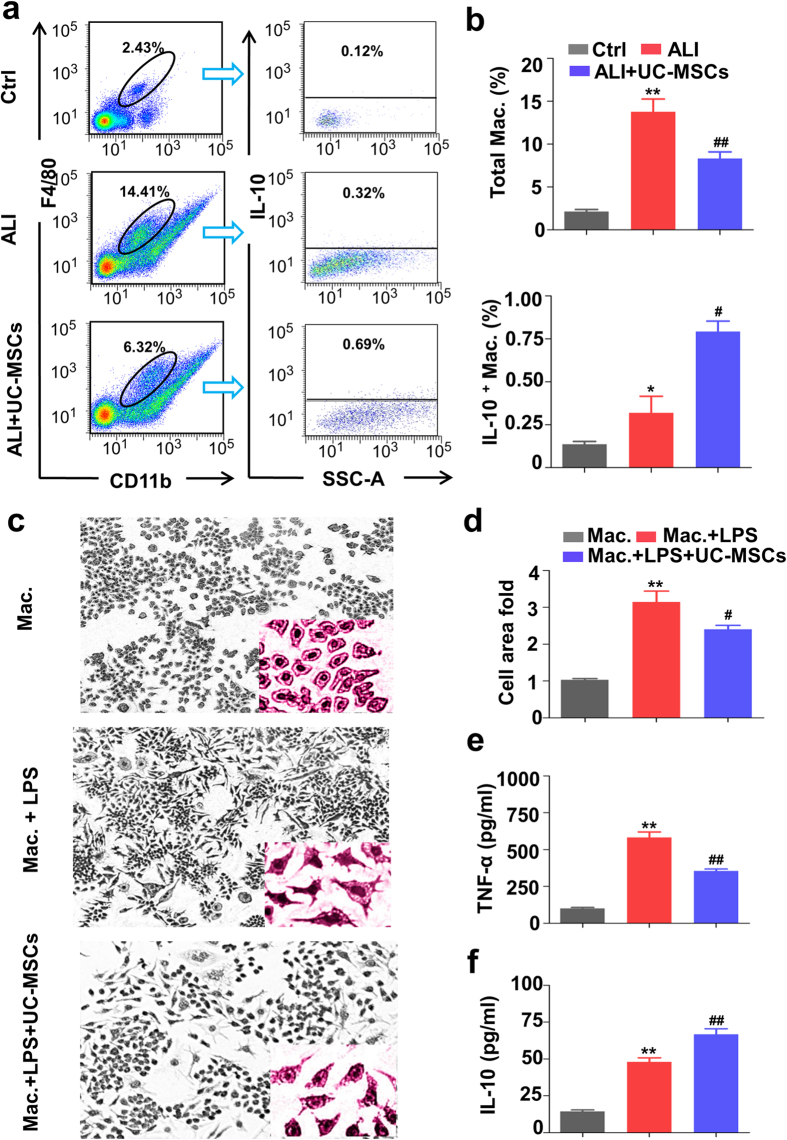
UC-MSCs modulate the immune response of macrophages in LPS-induced ALI mice. (**a**) The representative flow cytometric figures of lung CD11b^+^ F4/80^+^ and IL10^+^ macrophages. The lung tissues from LPS-induced ALI mice were harvested, grinded, filtered, and washed to obtain the monotypes. The monotypes were then incubated with anti-CD11b, anti-F4/80 or anti-IL-10 and were analyzed by flow cytometry. The cells were gated on anti-CD11b and anti-F4/80 simultaneously, and followed by gating on anti-IL-10. (**b**) The statistical analysis of lung CD11b^+^-F4/80^+^ macrophages and IL10^+^ macrophages were performed by flow cytometry. (**c**) The morphologic alterations of mouse macrophage RAW264.7 cells were stimulated with LPS and were co-cultured with UC-MSCs, resulting in enlarged cell body, irregular shape, and a large number of pseudopodia. (**d**) The statistical average surface area of a single macrophage was analyzed by Image Pro-Plus (IPP) 6.0 software, and the cell surface area in macrophage Raw264.7 cells was taken as 1.0 fold. The secretion concentration of TNF-α (**e**) and IL-10 (**f**) was measured by ELISA. Values are the means ± SE. n = 6–8 for each group. *p < 0.05 or **p < 0.01 *vs.* control mice (Ctrl) or macrophages. ^#^*p* < 0.05 or ^##^*p* < 0.01 *vs.* LPS-induced ALI mice (ALI) or LPS-stimulated macrophages. Mac., macrophages.

To explore the effects of UC-MSCs on macrophage transition, we examined the lung macrophages in UC-MSCs-treated ALI mice by flow cytometry. The results demonstrated that the total number of lung macrophages (Maker, F4/80 and CD11b) was significantly reduced in UC-MSCs-treated ALI mice, but the IL-10 positive lung macrophages (Maker, IL-10) were up-regulated (Fig. 3a,b, *p* < 0.05), indicating that UC-MSCs modulate the immune response of lung macrophages in ALI mice by promoting lung macrophages to secret more IL-10 to protect ALI mice. Therefore, UC-MSCs might alleviate the inflammatory response in ALI mice by mediating macrophages transition.

We then co-cultured LPS-stimulated mouse macrophage RAW264.7 cells with UC-MSCs to verify their relationship *in vitro*. The morphology of macrophages was significantly altered by LPS stimulation, including an enlarged cell body, irregular shape, and a large number of pseudopodia. Interestingly, when co-cultured with UC-MSCs, the morphology of LPS-stimulated RAW264.7 cells was presented as normal RAW264.7 cells (Fig. 3c,d). In addition, TNF-α expression in LPS-stimulated macrophages was significantly decreased by UC-MSCs co-culture, while IL-10 expression was increased (Fig. 3e,f), indicating that UC-MSCs promote the M2 polarization of macrophages.

### UC-MSCs protect LPS-induced ALI mice *via* paracrine activity

**Figure 4:**
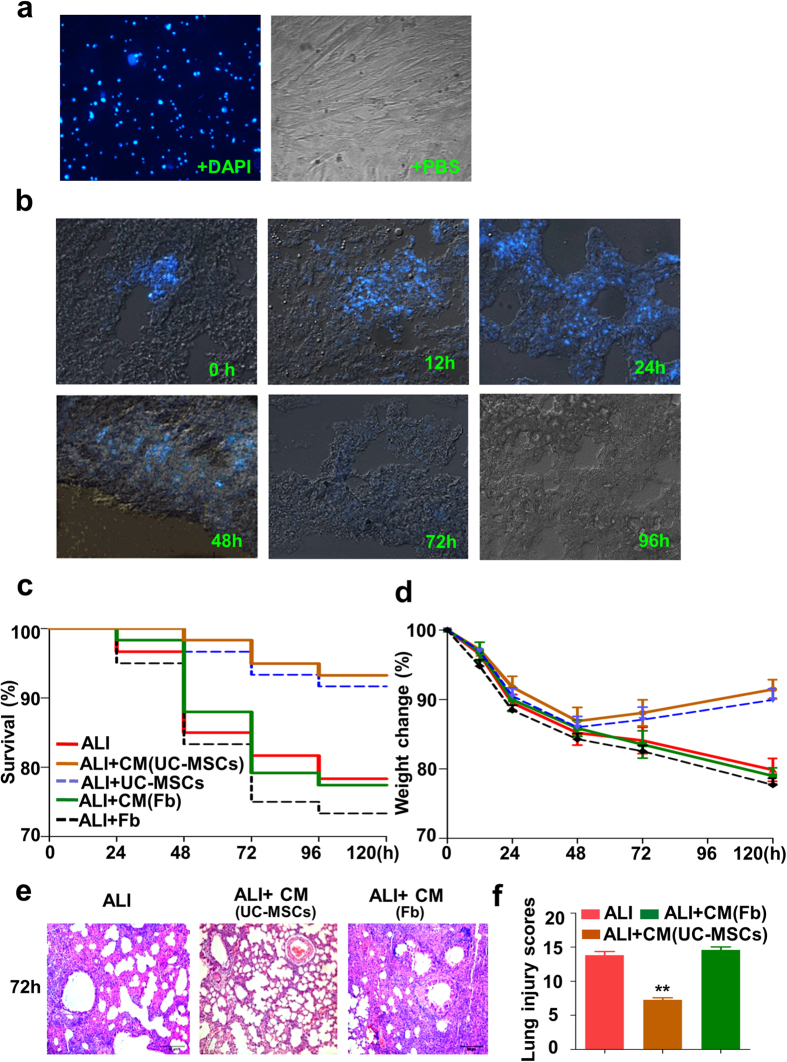
UC-MSCs ameliorate the lung injury of LPS-induced ALI mice through paracrine role. (**a**) Representative images of UC-MSCs stained with DAPI and imaged by fluorescence microscopy (left panel) or by light microscopy (right panel). (**b**) The fluorescence images of DAPI-stained UC-MSCs in lung cryosections from LPS-induced ALI mice at different post-delivery times. After UC-MSCs were intratracheally delivered, DAPI-labeled UC-MSCs were presented along the bronchial wall at post-deliver 0 h, they were accumulated in lung tissue 12 h–24 h post-delivery, rapidly decreasing in number 48 h post-delivery, and becoming rare 72 h to 120 h post-delivers. Magnification, ×100. n = 24 each group. (**c–f**) Injection of conditional medium (CM) of UC-MSCs effectively protects LPS-induced ALI mice. Kaplan-Meier survival curves (**c**) and body weight alteration (**d**) of mice, n = 24 for each group. HE staining of lung sections (**d**) and lung injury scores (**e**) in LPS-induced ALI mice treated with CM from the same amount of UC-MSCs or fibroblasts (Fb). Magnification, ×100. In (**f**), Values are the means ± SE. n = 7–9 for each group. **p < 0.01, LPS-induced ALI mice *vs.* LPS-induced ALI mice treated with conditional medium from UC-MSCs. CM, conditional medium.

Given that MSCs generate their therapeutic action through cell engraftment differentiation to directly repair injured tissues or through paracrine to facilitate self-healing of tissues^20^, we labeled UC-MSCs with DAPI *in vitro* and then tracked the labeled UC-MSCs in ALI mice post-injection at different times. The results showed that UC-MSCs were concentrated in the trachea 0 h post-injection, continuously infiltrated the lung parenchyma though the tracheal wall 12 h post-injection, and populated the whole lung parenchyma 24 h post-injection. However, UC-MSCs were still localized in the lung parenchyma 48 h post-injection, but the intensity of fluorescence was much lower than that of 24 h post-injection. Additionally, the DAPI-labeled UC-MSCs almost disappeared in the fluorescent microscope 72 h post-injection (Fig. 4a,b), which indicated that the time of UC-MSCs engraftment and therapeutic effects was 24 h–48 h post-injection, suggesting that UC-MSCs secrete soluble factors responsible for relieving ALI mice, rather than differentiating target cells and replacing.

To further confirm the paracrine activity of UC-MSCs in LPS-induced ALI mice, we applied the concentrated conditioned medium from UC-MSCs to treat LPS-induced ALI mice. The results demonstrated that the conditional medium of UC-MSCs could improve the survival and body weight (Fig. 4c,d), and ameliorate the lung injury of LPS-induced ALI mice (Fig. 4e,f), indicating that UC-MSCs secrete soluble factors responsible for ALI mice treatment.

### UC-MSCs protect ALI mice by secretion of PGE2

MSCs are reported to release multiple paracrine factors, especially prostaglandin-E2 (PGE2), secreted from BM-MSCs, with powerful immunological regulation functions^21^, which cause macrophages to secrete IL-10 to accelerate recovery of damaged tissues^22^,^23^. However, little is known about the ability of paracrine PGE2 in UC-MSCs. We analyzed the data of the gene expression array (GSE48022) from the GEO database and found that the gene expression of prostaglandin-endoperoxide synthase 2 (PTGS2/Cox-2), the rate-limiting enzyme in the biosynthesis of PGE2, is much higher in UC-MSCs than that in BM-MSCs (Suppl. Fig. 4a), indicating the powerful ability of synthesis and secretion of PGE2 in UC-MSCs. To further verify the secretion ability of PGE2 and its biological function, we analyzed the concentration of the small molecules in the UC-MSCs conditional medium, and found that the expression of PGE2 was several folds higher than that in LPS-stimulated macrophage medium. In addition, when co-cultured with LPS-stimulated macrophages, the production of PGE2 in UC-MSCs was significantly enhanced (Suppl. Fig. 4b), suggesting that the key role of PGE2 secreted by UC-MSCs for macrophages transition.

To confirm the effect of PGE2 secreted by UC-MSCs on macrophages, we applied celecoxib, a selective inhibitor of PGE2 biosynthesis, to approve the role of PGE2 in UC-MSCs-treated macrophages and mice. The results showed that a 1 μmol/L concentration of celecoxib significantly reduced PGE2 expression in UC-MSCs without drug toxicity (Suppl. Fig. 4c,d). Then we harvested the 1 μmol/L celecoxib-pretreated UC-MSCs (UC-MSCs^−PGE2^), which PGE2 synthesis in cells was inhibited, and the conditional medium form celecoxib-pretreated UC-MSCs (UC-MSCs^−PGE2^), which PGE2 concentrations was reduced. And we added them into LPS-stimulated macrophages and measured the TNF-α and IL-10 concentrations in the culture medium. The results showed that both the celecoxib-pretreated UC-MSCs (UC-MSCs^−PGE2^) and the conditional medium from celecoxib-pretreated UC-MSCs (CM^−PGE2^) had a weaker capacity for inhibiting TNF-α (Suppl. Fig. 4e) and stimulating IL-10 (Suppl. Fig. 4f) in LPS-stimulated macrophages, indicating that UC-MSCs increased PGE2 synthesis and secretion when interacting with LPS-stimulated macrophages, resulting in the inhibitory effects on TNF-α and stimulatory effects on IL-10.

**Figure 5:**
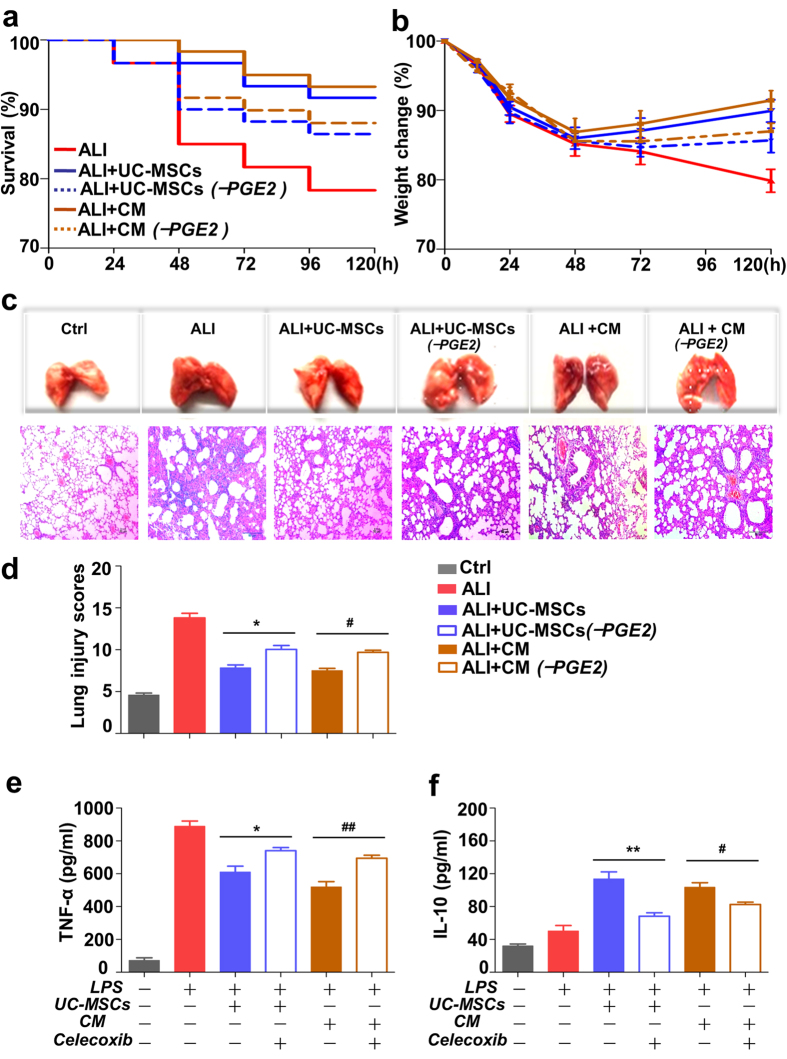
PGE2 secreted by UC-MSCs ameliorate the lung injury of LPS-induced ALI. Kaplan-Meier survival curves (**a**), body weight change (**b**), HE staining of lung sections (**c**), and lung injury scores (**d**) of LPS-induced ALI mice with a variety of treatments. The protection of UC-MSCs and UC-MSCs conditional medium (CM) on mice was weakened by reduced PGE2 levels. n = 24 for each group. Magnification, ×100. The concentration of TNF-α (**e**) and IL-10 (**f**) detected by ELISA in the lung BALF of mice with several treatments. In (**c**–**e**), values are the means ± SE. n = 7–9 at each point. *p < 0.05 or **p < 0.01, LPS-induced ALI mice treated with normal UC-MSCs (ALI + UC-MSCs) *vs.* LPS-induced ALI mice treated with celecoxib-pretreated UC-MSCs (ALI + UC-MSCs^−PGE2^). ^#^*p* < 0.05 or ^##^*p* < 0.01, LPS-induced ALI mice treated with conditional medium from normal UC-MSCs (ALI + CM) *vs.* LPS-induced ALI mice treated with conditional medium from celecoxib-pretreated UC-MSCs (ALI + CM^−PGE2^).

To further investigate the importance of PGE2 expression in the treatment of UC-MSCs in ALI mice, we treated mice with the celecoxib-pretreated UC-MSCs (UC-MSCs^−PGE2^) or the conditional medium from celecoxib-pretreated UC-MSCs (CM^−PGE2^) and observed the lung pathological alterations and the concentration of soluble factors in the BALF of these mice. The results (Fig. 5a–f) showed that the therapeutic effect of UC-MSCs on LPS–induced ALI mice declined with the decrease of PGE2 production, demonstrating that the rapid high expression of PGE2 secreted by UC-MSCs mediated the protection of UC-MSCs on LPS-induced ALI mice.

### The paracrine molecules of UC-MSCs in relief of ALI

**Figure 6:**
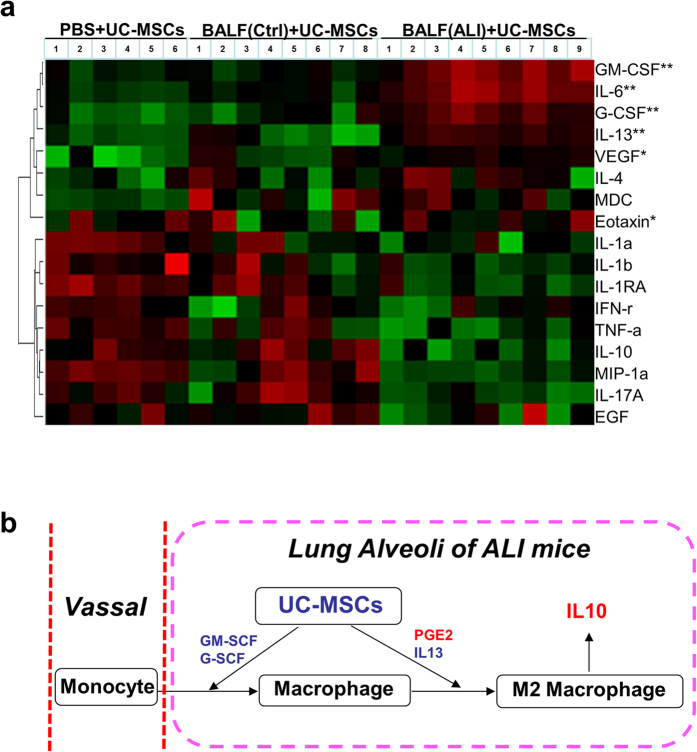
The multiple factors secreted by UC-MSCs may contribute to lung injury amelioration. (**a**) The paracrine factors of UC-MSCs were analyzed by using Multiple-ELISA assay and expressed as a heatmap. The increased proteins are shown in progressively brighter shades of red, and decreased proteins are shown in progressively darker shades of green. ^#^*p* < 0.05 or ^##^*p* < 0.01, UC-MSCs + BALF (ALI) *vs*. UC-MSCs + BALF (Ctrl), n = 8–9. (**b**) The scheme of factors secreted by UC-MSCs in ALI amelioration.

To further investigate the paracrine secretions of UC-MSCs in this mouse model of ALI, we found that a panel of the molecules secreted by the stem cells was increased significantly when cultured in BALF from ALI mouse compared with control mice (Fig. 6a). This indicated that UC-MSCs could secrete a group of paracrine (including GM-CSF, IL-6 and IL-13) responsible for ameliorating ALI in our mouse model. All the paracrine molecules are summarized in Fig. 6b.

## Discussion

In this study, we illustrated that intratracheal injection of UC-MSCs increased the survival rate of LPS-induced ALI mice and significantly reduced pulmonary inflammation. The soluble factors secreted by UC-MSCs, in particular, PGE2, GM-CSF, IL-6 and IL-13 may be the therapeutic basis of UC-MSCs for ALI.

ALI usually develops in patients with predisposing conditions that induce systemic inflammatory response, such as sepsis, pneumonia, major trauma, multiple transfusions, aspiration, and acute pancreatitis^24^. Among them, sepsis is the major etiology for ALI development, wherein Gram-negative bacteria are a prominent cause^25^. LPS, the main component of the outer membranes of Gram-negative bacteria, is the most important antigen that promotes the development of ALI^26^. Therefore, the LPS-induced animal models could highlight ways to explore mechanisms of multiple diseases and provide useful information on the discovery of novel biomarkers and drug targets^27^. In our study, the animal model induced by intra-tracheal administration of LPS successfully duplicated human ALI. The LPS-induced ALI mouse model had a classical pulmonary inflammatory state of the lungs between 48 h and 120 h, but after the administration of UC-MSCs, a decreased lung pathological lesion and inflammatory response was observed at 72 h after UC-MSCs injection, including the lung MPO activity, total protein concentration, lung neutrophil infiltration intensity, the expression of various pro-inflammation cytokines in BALF and the immune response of LPS-induced ALI mice. The results were consistent with previous studies on the beneficial effects of BM-MSCs in resolving ALI^5^,^7^,^28^,^29^, proving that the intra-tracheal injection of UC-MSCs had protective effects in attenuating lung injury and inhibiting the lung inflammation of LPS-induced ALI mice.

Although there are therapeutic effects of MSCs for different ALI models^28^, the precise molecular mechanisms of MSCs action in ALI therapy are still unclear. Some studies found that BM-MSCs could mobilize into circulation in mice after LPS exposure, accumulate within the inflammatory site and differentiate to form endothelial and epithelium-like phenotype cells, like ACE1 and ACE2^29^,^30^. Some studies postulated that soluble factors secreted by BM-MSCs played a vital role in wound repair, most likely through their anti-inflammatory, anti-apoptotic, angiogenic and immunomodulatory properties^31^,^32^. To determine the UC-MSCs’ location and their potential action principles, we located the DAPI-labeled UC-MSCs in ALI mice and found that UC-MSCs concentrated in the mouse lung within 24 h–48 h. Such a short period of UC-MSCs action suggested that paracrine is the dominant mechanism rather than cell differentiation and replacement in ALI repair. This hypothesis was further confirmed by UC-MSCs conditional medium ameliorating LPS-induced ALI mice. Our results revealed that the paracrine capacity of UC-MSCs had a primary functional and survival benefit in LPS-induced ALI. The secreted soluble factors in the UC-MSCs conditional medium are key for relieving ALI.

PGE2, one of the UC-MSCs paracrine factors, has demonstrated beneficial effects in LPS-induced ALI mice, which could modulate macrophage responses and attenuate the LPS-induced inflammation. Previous studies demonstrated that an intravenous injection of BM-MSCs can beneficially modulate the host immune response, by increasing the release of PGE2 from the BM-derived MSCs, acting on the EP2 and EP4 receptors of the macrophages, increasing the production of IL-10 in the macrophages and reducing inflammation^22^,^23^,^33^. Here, we found that UC-MSCs secrete a large amount of PGE2; however, with the decline of the PGE2 level in UC-MSCs and UC-MSCs conditional medium, both showed a lower ability to promote the production of IL-10 in LPS-stimulated RAW264.7 cells and in LPS-induced ALI mice. The increasing IL-10 level in the lungs suppresses LPS-induced ALI by reducing induction of pro-inflammatory mediators and chemokines, reducing mast-cell activation and NF-κB activation, and down-modulating pathways generating oxidative stress^34^. Activation of MSCs impacts their therapeutic abilities in lung injury by increasing IL-10 and IL-1α levels. Thus, our data suggested that UC-MSCs-secreted PGE2 mediates the protective function of UC-MSCs in lung injury.

Moreover, we observed that UC-MSCs cultured in BALF from ALI mouse could have a high-level expression of some paracrine including GM-CSF, G-CSF, IL-6, IL-13 and others, as shown in Fig. 6a. Studies found Both GM-CSF and G-CSF belong to the family of colony-stimulating factors, playing a key role in host defense by regulating proliferation and differentiation of granulocytes and mononuclear phagocytes^35^. In response to pulmonary infection, local delivery of GM-CSF could increase the number of alveolus exudate macrophages derived from blood monocytes, to improve the host defense against microbial pathogens^36^. During recently years, the benefits of GM-CSF application have been demonstrated to protect lung injury in sepsis^37^. As for IL-6, it is a multifunctional cytokine involved in the regulation of complex cellular processes such as proliferation and differentiation, and act as a key player during immune and inflammation response^38^. Recently, IL-6 is found to promote survival of human CD11b^+^ peripheral blood mononuclear cells and induce M2-type macrophage polarization^39^. In addition to IL-6, the capacity of IL-13 on the transformation of macrophages into M2 macrophages had been confirmed in many previous studies^40^. Importantly, M2 macrophages are considered to play an essential role in regulation of immune response and resolution of lung inflammation^41^. Furthermore, studies also found that MSCs could induce M2 macrophages polarization through other soluble factors, such as TGF-β1^42^, TSG-6^43^ and IDO^44^, which could get together to promote disease amelioration^45^. Therefore, we deduced that UC-MSC could modulate immune responses of macrophages against LPS-induced lung injury not just by PGE2, but rather *via* a panel of secretory factors, such as GM-CSF, IL-6 and IL-13 also contributed to the treatment of ALI mice.

In conclusion, our results demonstrated that UC-MSCs significantly attenuate inflammatory responses and lung injury in LPS-induced ALI mice. The protective effect on ALI was due to secreted a panel of secretory factors, suggesting that UC-MSCs could be a novel therapeutic choice for ALI.

## Additional Information

**How to cite this article:** Zhu, H. *et al*. Therapeutic Effects of Human Umbilical Cord-Derived Mesenchymal Stem Cells in Acute Lung Injury Mice. *Sci. Rep.* **7**, 39889; doi: 10.1038/srep39889 (2017).

**Publisher's note:** Springer Nature remains neutral with regard to jurisdictional claims in published maps and institutional affiliations.

## Supplementary Material

Supplementary Information
