# Supplementary material for: Therapeutic Effects of Human Umbilical Cord-Derived Mesenchymal Stem Cells in Acute Lung Injury Mice
Source: Sci Rep. 2017 Jan 4;7:39889. doi: 10.1038/srep39889 (PMC5209685; doi:10.1038/srep39889)
Supplement: Supplementary Information [file srep39889-s1.pdf]

Therapeutic Effects of Human Umbilical Cord-Derived Mesenchymal Stem Cells in Acute Lung Injury Mice

Hua Zhu[1, 2*]; Yi Xiong[1*]; Yunqiu Xia[1, 4]; Rong Zhang[1]; Daiyin Tian[1, 4]; Ting Wang[1, 4]; Jihong Dai[1, 4]; Lijia Wang[1]; Hongbing Yao[1, 3]; Hong Jiang[2]; Ke Yang[1, 6]; Enmei Liu[1, 4]; Yujun Shi[7]; Zhou Fu [1, 4, 6]; Li Gao [1,3, 6†]; Lin Zou [1, 5, 6†]

[1]Pediatrics Research Institute, Ministry of Education Key Laboratory of Child Development and Disorders, Children's Hospital of Chongqing Medical University, Chongqing 400014, China; [2]Department of Pediatrics, First Affiliated Hospital of China Medical University, Shenyang 110001, China; [3]Department of Otorhinolaryngology, Children's Hospital of Chongqing Medical University, Chongqing 400014, China; [4]Department of Respiratory Medicine, Children's Hospital of Chongqing Medical University, Chongqing 400014, China; [5]Center for Clinical Molecular Medicine, Children's Hospital of Chongqing Medical University, Chongqing 400014, China; [6]Chongqing Engineering Research Center of Stem Cell Therapy, Chongqing 400014, China; [7]Laboratoryof Pathology, West China Hospital, Sichuan University, Chengdu 610041, China;

[*] Authors are equally contributed to this work;

[†]**Address Co-correspondence to:**

Li Gao, 136 Zhongshan Er Road, Yuzhong District, Chongqing400014, China

Telephone: 86-23-63633054; Fax: 86-23-63622874; Email: 416875725@qq.com

Lin Zou, 136 Zhongshan Er Road, Yuzhong District, Chongqing 400014, China

Telephone: 86-23-63622128; Fax: 86-23-63622001; Email: zoulin74@126.com.

1.Supplementary Methods

LPS-induced ALI mice model

Eight- to ten-week-old BALB/c and C57BL/6 female mice were obtained from the Experimental Animal Center of Chongqing Medical University. All the experiments were approved by the Ethics Committee of Chongqing Medical University. The LPS-induced ALI model was performed in BALB/c and C57BL/6 mice, as previously described [1] and depicted the timeline for these experiments (Suppl.Fig.1a). Briefly, the mice were anesthetized by intraperitoneal administration of 10% chloral hydrate (30mg/kg) (Sigma-Aldrich, Missouri, USA). To choose an appropriate concentration of Lipopolysaccharide (LPS, Sigma-Aldrich, Missouri, USA), we intratracheally injected mice with 1.0, 2.0, 5.0, or 7.5 (mg/kg) LPS in 30μl sterile PBS or 30μl sterile PBS as the control with an indwelling vein needle. After 2h of LPS exposure, $0.5x10^6$ UC-MSCs (or the same amount of fibroblasts) in 80μl sterile PBS, 80μl concentrated UC-MSCs conditional medium, or 80μl sterile PBS as the control was administered intravenously to mice.

Culture and Characterization of UC-MSCs

The UC-MSCs isolated from human Wharton's Jelly were donated by Chongqing Engineering Research Center of Stem Cell Therapy. The UC-MSCs were cultured and characterized for expression of specific cell surface markers and differentiation to osteogenic, adipogenic, and chondrogenic cells, as previously reported [2-4]. The fifth passage (P5) of UC-MSCs were used in this study.

Histological evaluation of lung injury

Lung tissues were removed immediately after animals were sacrificed, and lung sections with hematoxylin and eosin (HE) staining were used to assess the severity of lung injury scores under light microscopy as the previous report [5].

Myeloperoxidase (MPO), protein levels and cell counts in BALF

Total cells and neutrophils were counted with a hemocytometer in a double-blind manner. Protein levels in the BALF supernatants were determined using the bicinchoninic acid protein assay (BCA) kit (Bioteke, Beijing, China). MPO activity was measured with an MPO assay kit (Nanjing Jiancheng Bioengineering Institute, Nanjing, China). All assays were performed according to the manufacturers' instructions.

Flow cytometry analysis of lung macrophages

The mice lungs were harvested and minced into small pieces. The pieces were incubated in RPMI 1640 medium (Gibco, Carlsbad, California, USA) with 0.1% collagenase type 1 (Sigma-Aldrich, Saint Louis, Missouri, USA) for 15min at 37°C and 5% CO_2. After incubation, the cell suspension was filtered through a 40μm cell strainer and was washed with PBS. The cells were stained with fluorescein monoclonal antibodies CD11b: FITC (BD, New Jersey, USA) and F4/80: APD (BD, New Jersey, USA), and were then processed with the Cytofix/Cytoperm™ Fixation/Permeabilization Solution kit (BD, New Jersey, USA), followed by incubation with IL-10: FITC (BD, New Jersey, USA) and analysis by flow cytometry. All protocols were performed according the manufacturers' instructions.

LPS-stimulated macrophages co-cultured with UC-MSCs or treated with UC-MSCs conditional medium *in vitro*

In 12-well tissue culture plates, 1.0x10^5 mouse macrophage RAW264.7 cells were cultured in DMEM with 10% FBS and were stimulated by LPS (1μg/mL) for 2h. The cells were then washed with PBS three times. Then, 1.0x10^5 normal UC-MSCs (or celecoxib-pretreated UC-MSCs) in 1ml DMEM/F12 with 10% FBS were added to the LPS-stimulated RAW264.7 cells, or 1ml serum-free conditional medium from normal US-MSCs (or from celecoxib-pretreated UC-MSCs) diluted 1:1 with fresh 20% FBS DMEM/F12 was added. The cells were incubated for 72 hours and the supernatants were collected for ELISA test.

DAPI staining for *in vivo* detection of UC-MSCs

Before inoculation, UC-MSCs were labeled with the nuclear stain 4', 6-diamidino-2-phenylindole dihydrochloride (DAPI) (Sigma-Aldrich, Missouri, USA). Briefly, DAPI was added to the culture medium for 30min when the cells were 70% confluent. Then, the cells were harvested using trypsinization and were prepared for intratracheal injection into lung with an indwelling vein needle. Lungs were harvested at 0h, 12h, 24h, 48h, 72h and 120h after UC-MSCs injection. Frozen lung specimens were cut and fixed in acetone. DAPI fluorescence was analyzed using fluorescence microscopy.

Quantitative real-time RT-PCR

Total RNA was isolated using TRIzol (Invitrogen, California, USA). The cDNA synthesis kit (Takara, Dalian, China) was applied to generate cDNA. Gene expression analysis was determined by quantitative real-time RT-PCR using the SYBR Green Mastermix (Tiangen, Beijing, China) and a q-PCR System (Bio-Rad, California, USA). The results were analyzed using the $2^{-\Delta\Delta CT}$ method with normalization against the inner

control glyceraldehyde 3-phosphate dehydrogenase (GAPDH) gene expression. The details of primers are shown in Suppl. Table 3.

ELISA test

The concentrations of mouse TNF-α and IL-10 (Neobioscience, Guangzhou, China) in animal BALF and expressed by LPS-stimulated macrophages RAW264.7, and human PGE2 (Enzo, New York, USA) secreted by UC-MSCs when cultured in normal conditional or co-cultured with LPS-stimulated RAW264.7 cells were measured by ELISA kits according to the manufacturers' instruction.

Analysis of Cox2 gene expression from a GSE27390 gene-chip

The gene expression profile of GSE48022 was downloaded from the GEO dataset, including 7 samples from BM-MSCs (n=4) and UC-MSCs (n=3), and gene expression of Cox2, the key synthesis enzyme for PGE2, was analyzed. The red plots showed the significantly differentially expressed genes between BM-MSCs and UC-MSCs, which were obtained with the thresholds of |logFC|>1.0 and Adj. P.V <0.05, using the t-test method in the LIMMA software package.

2.Supplementary Results:

Morphologic and characterized of UC-MSCs

UC-MSCs were effectively cultured and appeared as a firoblast-like or spindle-shaped morphology; And after 10th passages (P10), the UC-MSCs were still kept the cell morphology with rapidly proliferation ability without overt changes (Suppl. Fig. 1a-1b). The results of immunophenotypic flow cytometry of UC-MSCs showed that the cells were expressed almost all of the immune phenotypes with mesenchymal stem cells

(Suppl. Fig. 1c), including high level of stromal markers (CD73, CD90 and CD105) and low levels or absence of hematopoietic markers (CD34, CD45 and HLA-DR). Cells were investigated for UC-MSCs in adipogenic (Suppl. Fig. 1d-1), osteogenic (Suppl. Fig. 1d-2), and chondrogenic differentiation (Suppl. Fig. 1d-3) to show their multipotent differentiation capacity. Under adipogenic induction, UC-MSCs were presented with numerous lipid vacuoles that were detected by positive staining of Oil Red O. When cultured in osteogenic condition, the depositions of calcium in the cells were stained red by Alizarin Red S staining. And after induced by chondrogenic condition, the positive staining of Alcian blue indicated that UC-MSCs could differentiate into chondrocytes. Non-treated control cell showed no differentiation of adipocytes or osteoblasts or chondrocytes.

Establishing of LPS-induced ALI mice model

After evaluating the survival rate of ALI-mice induced by a different concentration of LPS (1.0 mg/kg; 2.5 mg/kg; 5.0 mg/kg; 7.5 mg/kg) since 24 h by intratracheally injected (Suppl. Fig. 2b). We found that 5.0mg/kg LPS could resulted in a remarkable pathological damage of lung tissue by HE staining (Suppl Fig 2c). Inaddtionly, the lung injury score of mice was the most serious at 72h after LPS injection, indicating that LPS-induced ALI mice model was successfully established for our subsequent experiments.

Reference

1. Alm, A.S., et al. Variation of lipopolysaccharide-induced acute lung injury in eight strains of mice. Respir Physiol Neurobiol **171**, 157-164 (2010).
2. Chen, M.Y., et al. Endothelial differentiation of Wharton's jelly-derived mesenchymal stem cells in comparison with bone marrow-derived mesenchymal stem cells. Exp Hematol **37**, 629-640 (2009).
3. Tong, C.K., et al. Generation of mesenchymal stem cell from human umbilical cord tissue using a combination enzymatic and mechanical disassociation method. Cell Biol Int **35**, 221-226 (2011).
4. Nekanti, U., et al. Long-term expansion and pluripotent marker array analysis of Wharton's jelly-derived mesenchymal stem cells. Stem Cells Dev **19**, 117-130 (2010).
5. Sakashita, A., et al. Neutrophil elastase inhibitor (sivelestat) attenuates subsequent ventilator-induced lung injury in mice. Eur J Pharmacol **571**, 62-71 (2007).

3. **Supplementary Figure Legends**

Suppl. Figure 1. Successful identification of UC-MSCs. (**a**) The morphologic features of UC-MSCs. The primary (P0) and early (P3) and late (P10) passages of cells were cultured and appeared fibroblast-like or spindle-shaped. (**b**) The cell proliferation of P3/P10 was analyzed by MTT assay, and the growth curves were drawn. (**c**) The flow cytometric images of the indicated cell surface markers of UC-MSCs, including CD34, CD45, CD73, CD90, CD105 and HLA-DR. The blue area represents antibody isotypes control for the background fluorescence, and the red area shows signal from the UC-MSCs surface marker antibodies. (**d**) The multi-lineage differentiation potential of UC-MSCs. UC-MSCs were cultured in inductive differentiation medium to show adipogenic differentiation as green arrows based on Oil Red O staining (**1**), osteogenic differentiation as red arrows based on alizarin red S staining (**2**), and chondrogenic differentiation as yellow arrows based on Alcian blue staining (**3**). UC-MSCs were cultured in basal medium as negative controls for staining with Oil Red O (**4**) and alizarin red S (**5**), and Alcian blue (**6**).

Suppl. Figure 2. Successful construction of a LPS-induced ALI mice model. (**a**) The work flow of our research. (**b**) Kaplan-Meier survival curves of BALB/C and C57B/L mice injected with LPS in different concentrations. n=24 for each group. HE staining (**c**) of lung sections and lung injury scores (**d**) from 5.0mg/kg LPS-induced ALI mice treated at different post-injection times. Magnifications, ×100 or ×400. Values are the means ± SE. n=10-12 at each point. $^{**}p<0.01$ *vs.* mice treated with PBS (Ctrl).

Suppl. Figure 3. UC-MSCs change the secreted factors from different macrophage

subtypes in the LPS-induced ALI mice. (**a**) The assembled factors secreted from M1 macrophage or M2 macrophage subtypes in LPS-induced ALI mice treated with UC-MSCs, detected by a RayBiotech™ 308 biotin label-based mouse antibody array and analyzed by the folds. M1 macrophage, classically activated macrophage. M2 macrophage, alternatively activated macrophage. (**b**) The relative mRNA fold of secreted factor gene from different macrophage subtypes in the LPS-induced ALI mice, which was normalized to the inner control GAPDH. The relative mRNA fold of LPS-induced mice was taken as 1.0 fold. In (**c**) to (**d**), the concentration of TNF-α and IL-10 in BALF, detected by ELISA from LPS-induced ALI mice treated with UC-MSCs. In (**b**) to (**d**), values are the means ± SE. n =8 for each group. $^{*}p<0.05$ or $^{**}p<0.01$ *vs.* control mice (Ctrl); $^{\#}p<0.05$ or $^{\#\#}p<0.01$ *vs.* LPS-induced ALI mice (ALI).

Suppl. Figure 4. PGE2 secreted from UC-MSCs inhibits inflammatory reaction of LPS-stimulated macrophages. (**a**) The mRNA expression data of PTGS2/Cox-2, the key synthesis enzyme for PGE2, in the GEO dataset (GSE48022) was significantly (LogFC=3.478, Adj. P.V.=0.018) higher in UC-MSCs than that in BM-MSCs. The gene expression profile of GSE48022 was downloaded from the GEO dataset, including 7 samples from BM-MSCs (n=4) and UC-MSCs (n=3). The red plots show the significantly differentially expressed genes between BM-MSCs and UC-MSCs, which were obtained with the thresholds of |logFC|>1.0 and Adj. P.V <0.05, using the t-test method in the LIMMA package. (**b**) The concentration of human PGE2 secreted by UC-MSCs cultured under normal condition or co-cultured with LPS-stimulated mouse macrophage RAW264.7 cells. (**c**) The expressing of human PGE2 secreted by UC-

MSCs when inhibited by different concentrations of celecoxib, which is the special inhibitor of COX-2 for PGE2 synthesis. Values are the means ± SE. n=6 for each group. (**d**) The cell proliferation measured by the MTT assay of UC-MSCs when inhibited by celecoxib in different concentrations. The secretion of TNF-α (**e)** and IL-10 (**f**) in LPS-stimulated macrophage RAW264.7 cells when co-cultured with celecoxib-pretreated UC-MSCs (UC-MSCs^{-PGE2}) or treated with conditional medium from celecoxib-pretreated UC-MSCs (CM^{-PGE2}). n=6-8 for each group，*p<0.05 or **p<0.01, LPS-stimulated macrophages co-cultured with normal UC-MSCs (Mac. + LPS + UC-MSCs) *vs.* LPS-stimulated macrophages co-cultured with celecoxib-pretreated UC-MSCs (Mac. + LPS + UC-MSCs^{-PGE2}). $^{\#}p$<0.05 or $^{\#\#}p$<0.01, LPS-stimulated macrophages treated with conditional medium from normal UC-MSCs (Mac. +LPS+ UC-MSCs) *vs.* LPS-stimulated macrophages treated with conditional medium from celecoxib-pretreated UC-MSCs (Mac. + LPS + CM^{-PGE2}). Mac., macrophage. CM, conditional medium. (**g)** The relative concentration fold of PGE2 by Elisa in different component. n=8 for each group. *p<0.05, fold of PGE2 in UC-MSCs Concentrated medium *vs.* that in UC-MSCs Original medium.

4.Supplementary Figures

Suppl.Fig.1

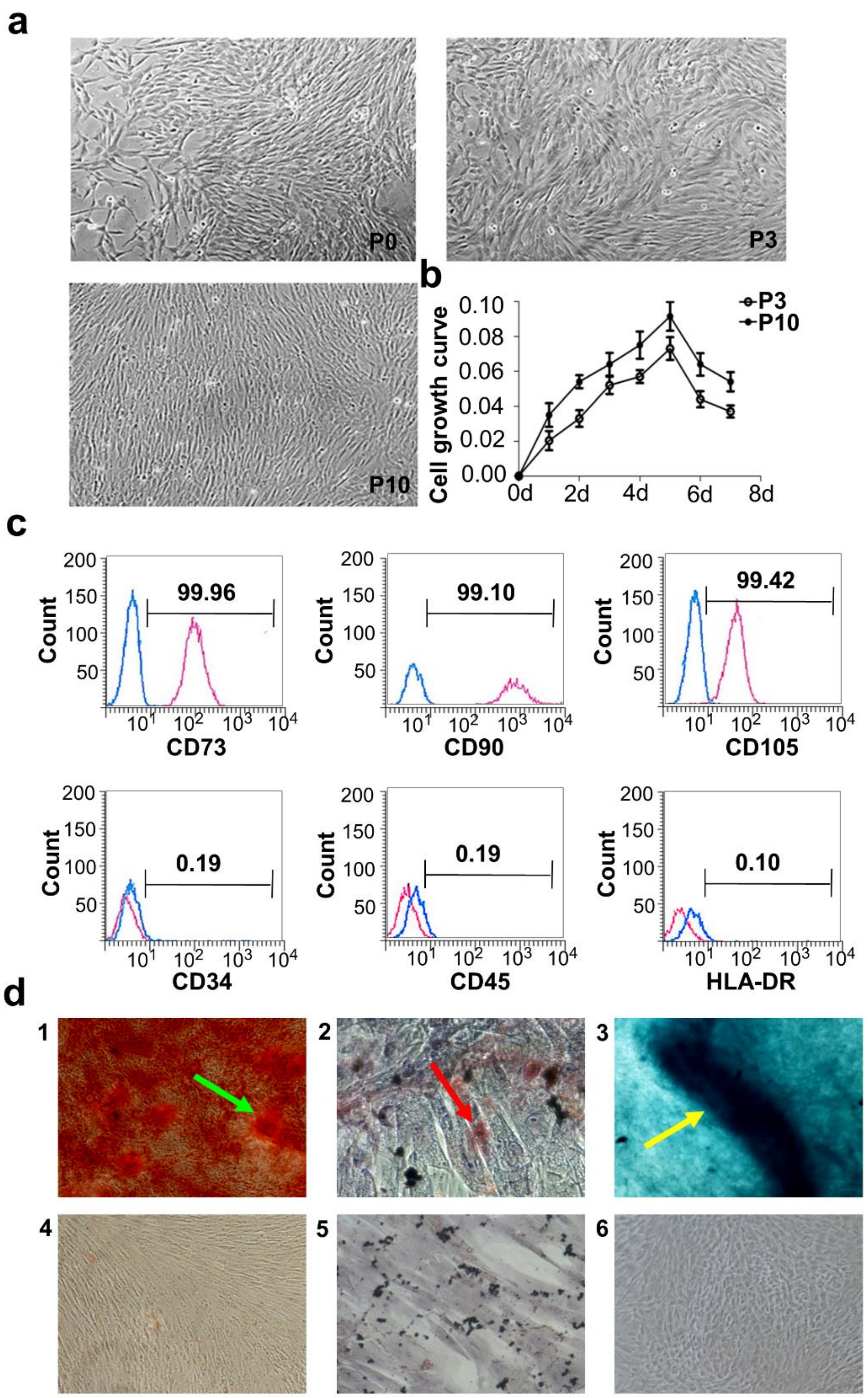

Suppl.Fig.2

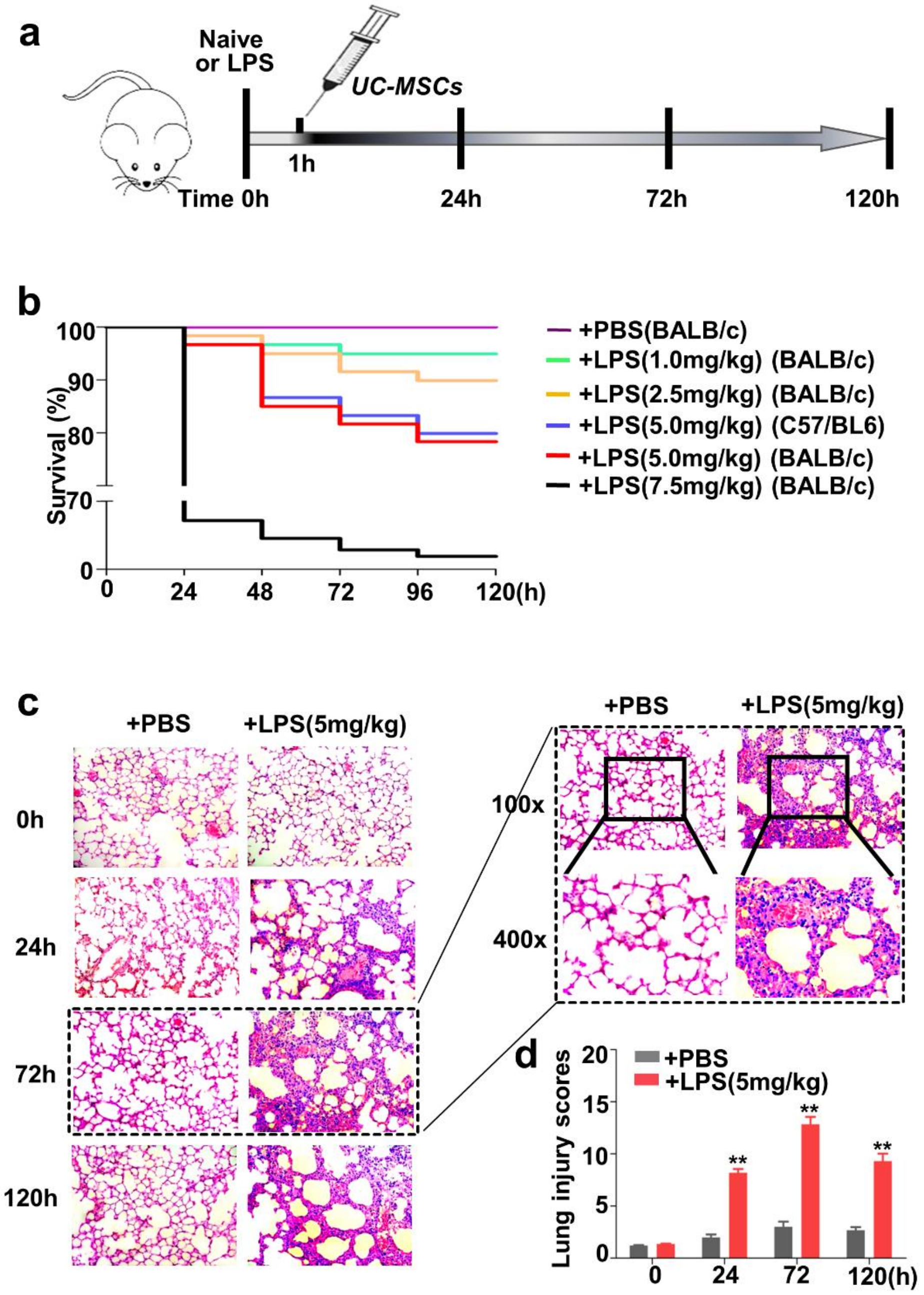

a
Naive
or LPS
UC-MSCs
1h
Time 0h
24h
72h
120h
b
100
90
80
70
0
Survival (%)
0
24
48
72
96
120(h)
+PBS(BALB/c)
+LPS(1.0mg/kg) (BALB/c)
+LPS(2.5mg/kg) (BALB/c)
+LPS(5.0mg/kg) (C57/BL6)
+LPS(5.0mg/kg) (BALB/c)
+LPS(7.5mg/kg) (BALB/c)
c
+PBS
+LPS(5mg/kg)
0h
24h
72h
120h
100x
400x
d
Lung injury scores
20
15
10
5
0
+PBS
+LPS(5mg/kg)
**
0
24
72
120(h)

Suppl.Fig.3

a

Classifications	Molecules	Mean (signal intensity) ALI (n=3)	Mean (signal intensity) ALI+US-MSC(n=3)	Fold ALI+US-MSCs/ALI
Associated with M1 macrophage	TNF-α	327.95	0.91	<0.01
	IL-1β	197.77	0.91	<0.01
	IL-6	1159.08	439.42	0.38
Associated with M2 macrophage	IL10	33.33	324.67	9.74
	CCL17	1.81	20.58	11.37
	CCL22	383.59	1289.48	3.36

b

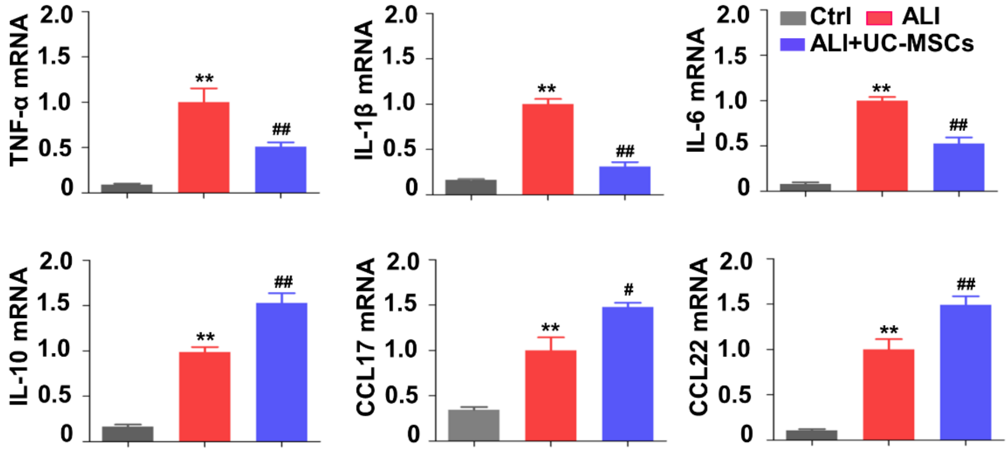

Suppl.Fig.4

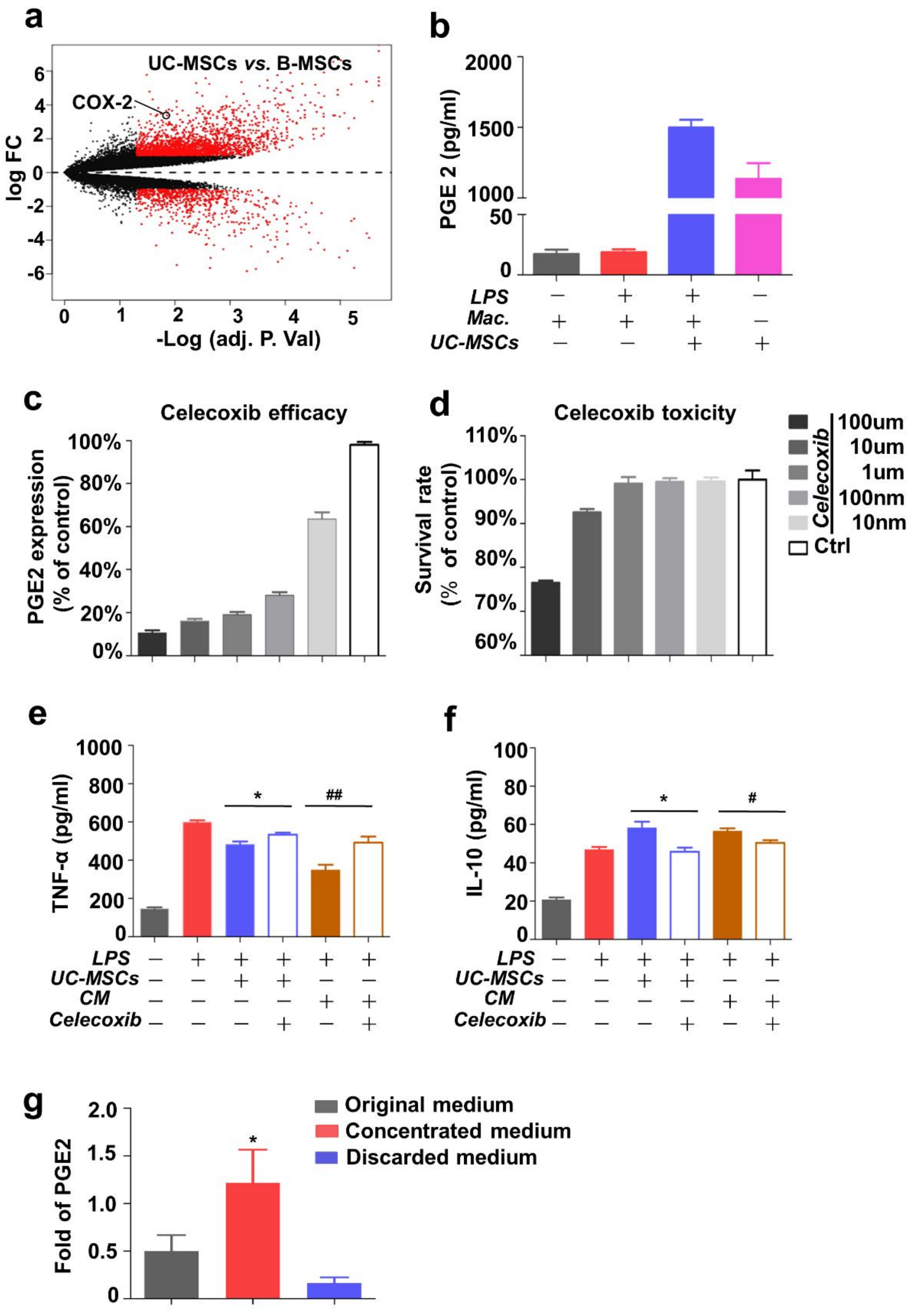
a
UC-MSCs vs. B-MSCs
COX-2
log FC
-Log (adj. P. Val)
b
PGE 2 (pg/ml)
LPS
Mac.
UC-MSCs
c
Celecoxib efficacy
PGE2 expression (% of control)
d
Celecoxib toxicity
Survival rate (% of control)
100um
10um
1um
100nm
10nm
Ctrl
Celecoxib
e
TNF-α (pg/ml)
LPS
UC-MSCs
CM
Celecoxib
f
IL-10 (pg/ml)
LPS
UC-MSCs
CM
Celecoxib
g
Fold of PGE2
Original medium
Concentrated medium
Discarded medium

5. Supplementary Tables

Suppl. Table 1: Protein expression profiles of BALF in LPS-induced ALI mice (ALI; n=3) and LPS-induced ALI mice treated with UC-MSCs (ALI+UC-MSCs; n=3).

Proteins	ALI	ALI+UC-MSCs	*p*-value	Fold
IL-1β	190.77	0.91	<0.01	<0.01
CCL27	285.05	0.91	<0.01	<0.01
CCL24	126.34	0.91	<0.01	0.01
TIMP-4	125.33	0.91	<0.01	0.01
IL-9R	129.57	0.91	<0.01	0.01
IGFBP-3	192.52	0.91	<0.01	0.00
AgRP	144.76	0.91	<0.01	0.01
DR3/TNFRSF25	76.61	0.91	<0.01	0.01
TGF-β3	568.44	21.38	<0.01	0.04
CCR9	90.88	0.91	0.01	0.01
CCL22	383.59	1289.48	0.01	3.36
TSLPR	170.38	0.91	0.01	0.01
IGF-1	251.59	0.91	0.01	0.00
IL-10	33.33	324.67	0.01	9.74
CXCL15	89.24	15.19	0.01	0.17
INHBC	73.10	0.91	0.01	0.01
TNF-α	327.95	0.91	0.01	0.00
IGFBP-1	110.37	0.91	0.02	0.01
TRAIL/TNFSF10	219.90	78.13	0.02	0.36
IL-6	1159.08	439.42	0.02	0.38
IGFBP-5	82.35	0.91	0.02	0.01
XCL1	95.15	0.91	0.02	0.01
CCL1/I-309/TCA-3	194.80	0.91	0.02	0.00
TIMP-2	1191.16	120.87	0.02	0.10
TLR3	185.50	0.91	0.02	0.00
TNFRSF19	291.58	0.91	0.02	0.00

IL-7	470.08	49.03	0.02	0.10
LIF	4.61	156.00	0.02	33.81
CXCL16	466.69	0.91	0.03	0.00
MIP-1γ	3237.27	4960.94	0.03	1.53
IL-17	711.11	72.17	0.03	0.10
PDGF-C	0.99	20.04	0.03	20.16
TLR1	231.67	0.91	0.03	<0.01
FADD	47.60	0.91	0.04	0.02
GM-CSF	99.11	0.91	0.04	0.01
TACI/TNFRSF13B	89.11	17.91	0.04	0.20
CCL17	1.81	20.58	0.04	11.36
IL-15	124.12	0.91	0.04	0.01
ICAM-1	1026.25	298.09	>0.05	0.29
ActivinA	145.33	0.91	>0.05	0.01
IL-17C	665.93	98.72	>0.05	0.15
IL-21	1.48	37.67	>0.05	25.37
IL-2	689.88	60.81	>0.05	0.09
FGF R3	122.50	0.91	>0.05	0.01
Kremen-2	50.30	16.04	>0.05	0.32
LRP-6	53.92	0.91	>0.05	0.02
TREM-1	267.65	94.43	>0.05	0.35
IL-17RD	98.53	212.11	>0.05	2.15
IL-9	134.03	0.91	>0.05	0.01
Lefty-1	16.12	0.91	>0.05	0.06
MMP-14/LEM-2	459.57	165.93	>0.05	0.36
VEGF-C	212.73	57.18	>0.05	0.27
TGF-β1	817.71	80.11	>0.05	0.10
TFPI	3.33	100.13	>0.05	30.06
TWEAK R / TNFRSF12	197.54	57.80	>0.05	0.29
IL-12p70	1639.79	344.01	>0.05	0.21
MFRP	173.74	50.50	>0.05	0.29
NGF R / TNFRSF16	189.55	50.50	>0.05	0.27

CCL28	108.22	0.91	>0.05	0.01
Insulin	76.15	22.23	>0.05	0.08
IL-1 R4 / ST2	92.59	0.91	>0.05	0.01
Urokinase	599.39	192.60	>0.05	0.32
uPAR	76.27	0.91	>0.05	0.01
Cerberus1	124.92	27.36	>0.05	0.22
6Ckine	40.70	10.69	>0.05	0.26
VEGF1	87.44	22.50	>0.05	0.26
FGF R5β	72.33	0.91	>0.05	0.01
IFN-β	1000.86	83.09	>0.05	0.08
VEGFR2	135.40	61.19	>0.05	0.45
IL-17F	11.38	0.91	>0.05	0.08
MCP-1	1873.59	632.95	>0.05	0.34
CRP	602.27	172.18	>0.05	0.29
IL-11	314.04	97.93	>0.05	0.31
IL-12Rβ1	247.56	67.82	>0.05	0.27
ICK	1804.97	24110.85	>0.05	13.36
IL-3	2157.47	481.18	>0.05	0.22
TMEFF1/Tomoregulin-1	76.60	9.35	>0.05	0.12
Pentraxin3/TSG-14	129.34	41.83	>0.05	0.32
KC	0.99	56.88	>0.05	57.21
NOV/CCN3	80.47	0.91	>0.05	0.01
TECK	181.76	71.32	>0.05	0.39
IL-1α	232.53	69.17	>0.05	0.30
CD40 Ligand/TNFSF5	9.59	0.91	>0.05	0.10
I-TAC	0.99	16.15	>0.05	16.25
Neuregulin-3/NRG3	46.23	5.64	>0.05	0.12
GFRα-4/GDNFRα-4	115.00	0.91	>0.05	0.01
AR (Amphiregulin)	9.07	0.91	>0.05	0.10
Fractalkine	11.21	0.91	>0.05	0.08
CD27/TNFRSF7	45.02	0.91	>0.05	0.02
CD27 Ligand / TNFSF7	25.30	0.91	>0.05	0.04

Leptin R	163.04	72.06	>0.05	0.44
bFGF	15.72	0.91	>0.05	0.06
Thymus Chemokine-1	229.20	114.77	>0.05	0.50
SCF	2.50	40.80	>0.05	16.34
Spinesin Ectodomain	131.64	0.91	>0.05	0.01
Epigen	46.01	0.91	>0.05	0.02
Adiponectin/Acrp30	32.93	0.91	>0.05	0.03
BLC	18.46	0.91	>0.05	0.05
TNF RII	1672.85	1107.80	>0.05	0.66
Angiopoietin-like 3	6.79	0.91	>0.05	0.13
GFRα-2/GDNF R α-2	24.16	0.91	>0.05	0.04
GDF-8	285.05	0.97	>0.05	0.00
S100A10	3.47	41.36	>0.05	11.93
GFRα-3/GDNFRα-3	18.87	0.91	>0.05	0.05
IL-17E	104.61	245.47	>0.05	2.35
IL-5Rα	42.34	0.91	>0.05	0.02
GITR Ligand/TNFSF18	712.48	160.36	>0.05	0.23
Osteoporotegerin	0.99	13.75	>0.05	13.83
EG-VEGF/PK1	708.01	230.93	>0.05	0.33
IL-2Rγ	296.85	125.67	>0.05	0.42
IL-21R	0.99	27.62	>0.05	27.78
IL-5	2235.13	645.13	>0.05	0.29
IFN-γ	1408.58	235.23	>0.05	0.17
Soggy-1	21.36	114.91	>0.05	5.38
SPARC	660.81	288.10	>0.05	0.44
VEGF-D	105.02	60.91	>0.05	0.58
IL-31	30.26	0.91	>0.05	0.03
IL-15 R α	163.40	57.17	>0.05	0.35
CD30 L	36.37	0.91	>0.05	0.03
IL-22BP	106.41	7.32	>0.05	0.07
Erythropoietin (EPO)	441.13	114.52	>0.05	0.26
Integrin β2/CD18	9.98	64.67	>0.05	6.48

IL-17D	30.11	114.24	>0.05	3.79
FCrRIIB/CD32b	71.95	0.91	>0.05	0.01
Neurturin	39.07	13.91	>0.05	0.36
TPO	107.89	31.21	>0.05	0.29
GDF-1	91.69	34.02	>0.05	0.37
Serum Amyloid A1	82.77	291.33	>0.05	3.52
PDGFRα	119.38	57.48	>0.05	0.48
IL-12p40/p70	249.26	0.91	>0.05	0.00
E-Selectin	67.32	0.91	>0.05	0.01
IL-2Rα	22.23	0.91	>0.05	0.04
CCR10	12.00	0.91	>0.05	0.08
IFN-α/β R1	14.61	0.91	>0.05	0.06
MMP-12	3.16	27.80	>0.05	8.79
IL-1 RII	32.36	0.91	>0.05	0.03
Shh-N	46.69	11.42	>0.05	0.24
PlGF-2	24.60	96.78	>0.05	3.93
CD11b	2515.73	1002.46	>0.05	0.40
IL-3Rβ	38.85	0.91	>0.05	0.02
HVEM/TNFRSF14	6309.81	4647.81	>0.05	0.74
IL-20 R α	61.49	146.22	>0.05	2.38
OX40 Ligand / TNFSF4	629.64	292.20	>0.05	0.46
IGFBP-rp1 / IGFBP-7	48.81	0.91	>0.05	0.02
Decorin	5.31	0.91	>0.05	0.17
MIP-3β	30.60	134.81	>0.05	4.41
ICAM-2/CD102	6.31	0.91	>0.05	0.14
MFG-E8	18.11	0.91	>0.05	0.05
TRAILR2 /TNFRSF10B	0.99	17.73	>0.05	17.84
Follistatin-like 1	684.12	307.61	>0.05	0.45
TNF-β/TNFSF1B	163.26	94.29	>0.05	0.58
IL-13	0.99	55.85	>0.05	56.17
IL-17BR	169.29	83.23	>0.05	0.49
IL-4R	8.70	0.91	>0.05	0.11

IL-7Rα	22.43	0.91	>0.05	0.04
IL-23	1.33	5.34	>0.05	4.02
TSLP	237.93	103.57	>0.05	0.44
CCR4	32.28	0.91	>0.05	0.03
CTLA-4/CD152	25.33	0.91	>0.05	0.04
IFN-α/β R2	1049.28	514.26	>0.05	0.49
Cripto	22.97	0.91	>0.05	0.04
IL-2Rβ	29.46	0.91	>0.05	0.03
HGFR	25.97	0.91	>0.05	0.04
CD30	18.65	0.91	>0.05	0.05
Angiopoietin-like 2	1.16	0.91	>0.05	0.79
SLPI	14954.97	11266.54	>0.05	0.75
DPPIV/CD26	12.15	0.91	>0.05	0.08
Crossveinless-2	2.47	0.91	>0.05	0.37
MMP-2	25.05	50.94	>0.05	2.03
Frizzled-7	29.80	0.91	>0.05	0.03
CCR3	2.63	0.91	>0.05	0.35
Dkk-4	18.82	0.91	>0.05	0.05
Artemin	2.96	0.91	>0.05	0.31
RAGE	0.99	17.20	>0.05	17.30
G-CSF	3.33	0.91	>0.05	0.27
IL-13Rα2	6.32	15.66	>0.05	2.48
Endostatin	3.94	0.91	>0.05	0.23
FAM3B	4.10	0.91	>0.05	0.22
IGFBP-2	4.43	0.91	>0.05	0.21
ALCAM	5.25	0.91	>0.05	0.17
CXCR3	4.83	0.91	>0.05	0.19
Epiregulin	4.83	0.91	>0.05	0.19
IL-22	217.73	166.58	>0.05	0.77
Growth Hormone R	5.66	0.91	>0.05	0.16
MMP-24/MT5-MMP	6.50	0.91	>0.05	0.14
BTC (βcellulin)	6.67	0.91	>0.05	0.14

CCR7	7.33	0.91	>0.05	0.12
CD14	7.33	0.91	>0.05	0.12
IGF-II	9.99	0.91	>0.05	0.09
IL-4	12.66	0.91	>0.05	0.07
CXCL14/BRAK	15.39	0.91	>0.05	0.06
GITR	13.99	0.91	>0.05	0.07
MIP-3 α	0.99	8.34	>0.05	8.38
Endoglin/CD105	16.16	0.91	>0.05	0.06
IGFBP-6	19.99	0.91	>0.05	0.05
Endocan	23.89	0.91	>0.05	0.04
VE-Cadherin	0.99	11.82	>0.05	11.89
TGF-βRI/ALK-5	0.99	15.53	>0.05	15.62
DKK-1	0.99	14.00	>0.05	14.08
P-Selectin	0.99	60.28	>0.05	60.63
Glut2	25.85	1.12	>0.05	0.04
MIP-1 α	0.99	17.46	>0.05	17.56
Resistin	320.77	187.56	>0.05	0.58
M-CSF	373.01	232.46	>0.05	0.62
WIF-1	0.99	7.95	>0.05	7.99
Dtk	750.54	368.07	>0.05	0.49
SDF-1	0.99	5.05	>0.05	5.08
Csk	4465.94	2702.71	>0.05	0.61
TLR4	5.16	32.51	>0.05	6.30
Granzyme B	398.15	207.28	>0.05	0.52
IL-20	80.54	63.00	>0.05	0.78
IL-31RA	44.74	68.35	>0.05	1.53
Thrombospondin	251.57	201.86	>0.05	0.80
L-Selectin	1377.07	1188.99	>0.05	0.86
MAdCAM-1	2.48	8.56	>0.05	3.45
IL-17R	131.58	76.23	>0.05	0.58
TWEAK/TNFSF12	174.37	115.66	>0.05	0.66
VEGFR3	14.48	28.79	>0.05	1.99

RANTES	0.99	1.74	>0.05	1.75
MCP-5	106.40	178.18	>0.05	1.67
Frizzled-6	135.85	78.27	>0.05	0.58
IL-18Rα/IL-1R5	371.90	535.00	>0.05	1.44
IL-17RC	170.04	119.52	>0.05	0.70
TCCR/WSX-1	273.37	186.52	>0.05	0.68
TL1A/TNFSF15	289.67	201.54	>0.05	0.70
MMP-3	31.76	53.49	>0.05	1.68
IL-27	655.31	1314.54	>0.05	2.01
IL-1R9	2257.13	1542.26	>0.05	0.68
TGF-β RII	559.52	369.67	>0.05	0.66
Galectin-3	1541.61	1252.64	>0.05	0.81
BCMA/TNFRSF17	0.99	0.91	>0.05	0.92
ICAM-5	0.99	0.91	>0.05	0.92
Activin RIB/ALK-4	0.99	0.91	>0.05	0.92
EGFR	0.99	0.91	>0.05	0.92
CCR6	0.99	0.91	>0.05	0.92
GDF-5	0.99	0.91	>0.05	0.92
CXCR4	0.99	0.91	>0.05	0.92
IL-10Rα	0.99	0.91	>0.05	0.92
CCL8/MCP-2	0.99	0.91	>0.05	0.92
Fit-3 Ligand	0.99	0.91	>0.05	0.92
Axl	0.99	0.91	>0.05	0.92
B7-1/CD80	0.99	0.91	>0.05	0.92
BAFFR/TNFRSF13C	0.99	0.91	>0.05	0.92
β-Catenin	0.99	0.91	>0.05	0.92
Cardiotrophin-1	0.99	0.91	>0.05	0.92
CCL4/MIP-1β	0.99	0.91	>0.05	0.92
CCL7/MCP-3 /MARC	0.99	0.91	>0.05	0.92
CD40	0.99	0.91	>0.05	0.92
Chordin-Like 2	0.99	0.91	>0.05	0.92
Tissue Factor	0.99	0.91	>0.05	0.92

CRG-2	0.99	0.91	>0.05	0.92
Cryptic	0.99	0.91	>0.05	0.92
CXCR2/IL-8 RB	0.99	0.91	>0.05	0.92
DAN	0.99	0.91	>0.05	0.92
Dkk-3	0.99	0.91	>0.05	0.92
EDAR	0.99	0.91	>0.05	0.92
Eotaxin	0.99	0.91	>0.05	0.92
Fas Ligand	0.99	0.91	>0.05	0.92
FGFR4	0.99	0.91	>0.05	0.92
FGF-21	0.99	0.91	>0.05	0.92
FLRG (Follistatin)	0.99	0.91	>0.05	0.92
Frizzled-1	0.99	0.91	>0.05	0.92
GDF-3	0.99	0.91	>0.05	0.92
GDF-9	0.99	0.91	>0.05	0.92
Granzyme D	0.99	0.91	>0.05	0.92
Granzyme G	0.99	0.91	>0.05	0.92
Gremlin	0.99	0.91	>0.05	0.92
HGF	0.99	0.91	>0.05	0.92
IL-1R	0.99	0.91	>0.05	0.92
IL-1R6/IL-1Rrp2	0.99	0.91	>0.05	0.92
IL-3R α	0.99	0.91	>0.05	0.92
IL-6R	0.99	0.91	>0.05	0.92
TGF-β2	0.99	0.91	>0.05	0.92
Ubiqultin	0.99	0.91	>0.05	0.92
LEPTIN(OB)	11.99	6.70	>0.05	0.56
IL-23R	1148.11	858.40	>0.05	0.75
LIGHT/TNFSF14	10.79	7.66	>0.05	0.71
MIG	289.33	210.82	>0.05	0.73
Fas/TNFRSF6	27204.87	25089.44	>0.05	0.92
WISP-1/CCN-4	127.43	99.01	>0.05	0.78
Kremen-1	48.14	60.71	>0.05	1.26
TRANCE/TNFSF11	604.53	457.91	>0.05	0.76

IFN-γR1	847.71	684.39	>0.05	0.81
TLR2	530.75	414.65	>0.05	0.78
Osteopontin	1523.80	1908.66	>0.05	1.25
MIP-2	728.90	623.73	>0.05	0.86
RELM β	14492.30	13954.90	>0.05	0.96
Tie-2	33.26	28.11	>0.05	0.85
TNFRI/TNFRSF1A	779.34	856.45	>0.05	1.10
IL-24	355.15	412.23	>0.05	1.16
PF-4	58.56	49.24	>0.05	0.84
CXCR6	876.60	782.39	>0.05	0.89
IL-28/IFN-lambda	148.75	171.65	>0.05	1.15
TIMP-1	583.31	608.00	>0.05	1.04
Osteoactivin / GPNMB	148.47	161.44	>0.05	1.09
VCAM-1	67.15	72.13	>0.05	1.07
LIX	240.93	252.19	>0.05	1.05
SIGIRR	101.05	105.36	>0.05	1.04
PDGFRβ	159.97	153.74	>0.05	0.96
TCA-3	384.34	364.26	>0.05	0.95
Progranulin	1885.92	1904.43	>0.05	1.01
Lymphotoxin βR/TNFRSF3	1279.53	1333.90	>0.05	1.04
IL-16	694.89	717.87	>0.05	1.03
VEGF	12.10	12.48	>0.05	1.03
SCFR/c-kit	636.39	645.32	>0.05	1.01
MMP-9	195.76	196.77	>0.05	1.01
Prolactin	562.68	564.90	>0.05	1.00
VEGF-B	673.76	674.12	>0.05	1.00

Suppl. Table 2: The analysis of Gene-ontology (GO) classifications on 37 differentially expressed proteins of BALF between LPS-induced ALI mice (ALI) and LPS-induced ALI mice treated with UC-MSCs (ALI+UC-MSCs).

Biological regulation	Immune response	Defense response	Macrophage
AgRp	CCL17	CCL17	CCL17
CCL22	CCL22	CCL22	CCL22
CCL24	CCL24	CCL24	IL-10
CCR9	CCL27	CCR9	IL-1β
CXCL16	CCR9	CXCL16	IL-6
FADD	CXCL16	IL-10	TNF-α
IGF-1	IL-10	IL-1β	
IGFBP3	IL-1β	IL-6	
IL-10	IL-6	TLR1	
IL-1β	IL-7	TLR3	
IL-6	LIF	TNF-α	
IL-7	TLR1		
IL-9R	TLR3		
LIF	TNF-α		
PDGFC	TNFRSF13B		
TGFB3	TNFSF10		
TIMP-2	XCL1		
TLR1			
TLR3			
TNF-α			
TNFRSF13B			
TNFRSF19			
TNFRSF25			
TNFSF10			
XCL1			

Suppl. Table 3: Primers for real-time RT-PCR

Genes		Primers
TNF-α	Forward	5’ CAGGCGGTGCCTATGTCTC 3’
	Reverse	5’ CGATCACCCCGAAGTTCAGTAG 3’
IL-1β	Forward	5’ GAAATGCCACCTTTTGACAGTG 3’
	Reverse	5’ TGGATGCTCTCATCAGGACAG 3’
IL-6	Forward	5’ CTGCAAGAGACTTCCATCCAG 3’
	Reverse	5’ AGTGGTATAGACAGGTCTGTTGG 3’
IL-10	Forward	5’ TTGTCGCGTTTGCTCCCATT 3’
	Reverse	5’ GAAGGGCTTGGCAGTTCTG 3’
CCL17	Forward	5’ TACCATGAGGTCACTTCAGATGC3’
	Reverse	5’ GCACTCTCGGCCTACATTGG 3’
CCL22	Forward	5’ AGGTCCCTATGGTGCCAATGT 3’
	Reverse	5’ CGGCAGGATTTTGAGGTCCA 3’
GAPDH	Forward	5’ GGTGAAGGTCGGTGTGAACG3’
	Reverse	5’ CTCGCTCCTGGAAGATGGTG 3’
